# Promising Cerebral Blood Flow Enhancers in Acute Ischemic Stroke

**DOI:** 10.1007/s12975-022-01100-w

**Published:** 2022-11-17

**Authors:** Ifechukwude Joachim Biose, Jadesola Oremosu, Somya Bhatnagar, Gregory Jaye Bix

**Affiliations:** 1 Department of Neurosurgery, Clinical Neuroscience Research Center, Tulane University School of Medicine, 131 S. Robertson, Ste 1300, Room 1349, New Orleans, LA 70112 USA; 2 School of Medicine, Tulane University, New Orleans, LA 70112 USA; 3 Tulane Brain Institute, Tulane University, New Orleans, LA 70112 USA; 4 Department of Neurology, Tulane University School of Medicine, New Orleans, LA 70112 USA; 5 Department of Microbiology and Immunology, Tulane University School of Medicine, New Orleans, LA 70112 USA; 6 School of Public Health and Tropical Medicine, Tulane University, New Orleans, LA 70122 USA

**Keywords:** Ischemic stroke, Cerebral blood flow, Collateral blood flow, Adjunctive therapy

## Abstract

Ischemic stroke presents a major global economic and public health burden. Although recent advances in available endovascular therapies show improved functional outcome, a good number of stroke patients are either ineligible or do not have access to these treatments. Also, robust collateral flow during acute ischemic stroke independently predicts the success of endovascular therapies and the outcome of stroke. Hence, adjunctive therapies for cerebral blood flow (CBF) enhancement are urgently needed. A very clear overview of the pial collaterals and the role of genetics are presented in this review. We review available evidence and advancement for potential therapies aimed at improving CBF during acute ischemic stroke. We identified heme-free soluble guanylate cyclase activators; Sanguinate, remote ischemic perconditioning; Fasudil, S1P agonists; and stimulation of the sphenopalatine ganglion as promising potential CBF-enhancing therapeutics requiring further investigation. Additionally, we outline and discuss the critical steps required to advance research strategies for clinically translatable CBF-enhancing agents in the context of acute ischemic stroke models.

## Introduction

Stroke is the global leading cause of debilitating long-term disability and accounts for approximately 6 million deaths per annum [1, 2]. The incidence of stroke is a major public health concern. Indeed, stroke occurs every 40 s and the yearly economic burden is over $45 billion in the USA alone [2]. Of all stroke cases, approximately 87% are due to ischemic stroke. Ischemic stroke occurs when blood vessels supplying the brain are occluded, cutting off oxygen and nutrients to brain cells in affected region(s). Timely restoration of cerebral blood flow (CBF) is the only established effective treatment for acute ischemic stroke. Recombinant tissue plasminogen activator (rt-PA), a thrombolytic drug, increases arterial reperfusion rates, restores perfusion, and improves functional outcomes [3]. Also, a number of recent clinical trials have demonstrated the efficacy of mechanical thrombectomy (MT), both as a lone treatment and in combination with rt-PA, in improving outcomes in the most severe cases of ischemic stroke with proximal large artery occlusion [4–8].

Despite these notable developments in the clinical management of acute ischemic cases, there remains a considerable need for alternative and adjunctive treatments. While most clinical centers have upgraded the therapeutic window for MT to 24 h from symptom onset, only a minor proportion of ischemic stroke patients are eligible for this treatment [9]. Half of the patients continue to experience ongoing symptoms or disability despite early recanalization, and reperfusion itself can cause brain injury [10, 11]. Indeed, rt-PA affords 30% early recanalization while the estimates of early recanalization following MT can reach 85% [11]. Safe adjunctive therapeutics which can be administered acutely to enhance CBF while stroke patients are in transit for expert medical intervention and/ or clinical evaluation and treatment is desirable and would increase the therapeutic time window by extending the life span of the ischemic penumbra—the viable hypo-perfused brain tissue which is the target of ischemic stroke treatments.

Previous clinical trials [12] for otherwise promising adjunctive therapies have failed, partly due to ambitious outcome measures. However, emerging opinions are increasingly pointed at the publication, quality, and reporting of preclinical evidence which birthed majority of the neutral clinical trials aimed at safety and efficacy in humans. It is well documented that until recently, the majority of journals favored preclinical findings which reported positive results and often rejected manuscripts showing neutral or negative results, even when the rationale and experimental design is sound [13, 14]. This phenomenon is described as publication bias. With increasing awareness of this pervasive problem, some journals have begun to accept manuscripts on the merit of their rationale and soundness of the science. This recent development will herald transparency of scientific reporting of neutral results as well as the documentation of limitations therein for further investigations. Also, majority of the potential therapies advanced to clinical trials often emanated from single-center reports. Some of the foundation preclinical studies did not consider randomizing animals to group before the commencement of the studies and the majority did not report blinding of the experimenters to test articles or group identifications during experimentation and/or data analysis. To improve the quality of preclinical evidence, forestall unconscious bias and increase reproducibility, the STAIR (Stroke Therapy Academic Industry Roundtable) recommendations [15] led to the more appropriate framing of the methodology and it is a major source of advocacy for multi-center experimentation, randomization, blinding, and defining exclusion criteria for animals without infarcts. Indeed, we believe that a continuous retrospective analysis of publications on preclinically tested potential adjunctive treatments reported to increase the effectiveness of recanalization treatments, ameliorate reperfusion injuries as well as enhance CBF through increased collateral flow should be weighed on the proverbial scale of the STAIR recommendations before being considered for clinical trial evaluation.

Here, we highlight the importance of collateral flow and the role of genetics in acute ischemic stroke. We review the potential pharmacological and adjunctive approaches reported to enhance CBF in experimental and clinical acute ischemic stroke settings. Next, we discuss the evidence and limitations of these “would be” therapies in the context of their relevance to clinical stroke comorbidities and functional recovery.

### Importance of Collateral Circulation in Acute Ischemic Stroke

**Fig. 1:**
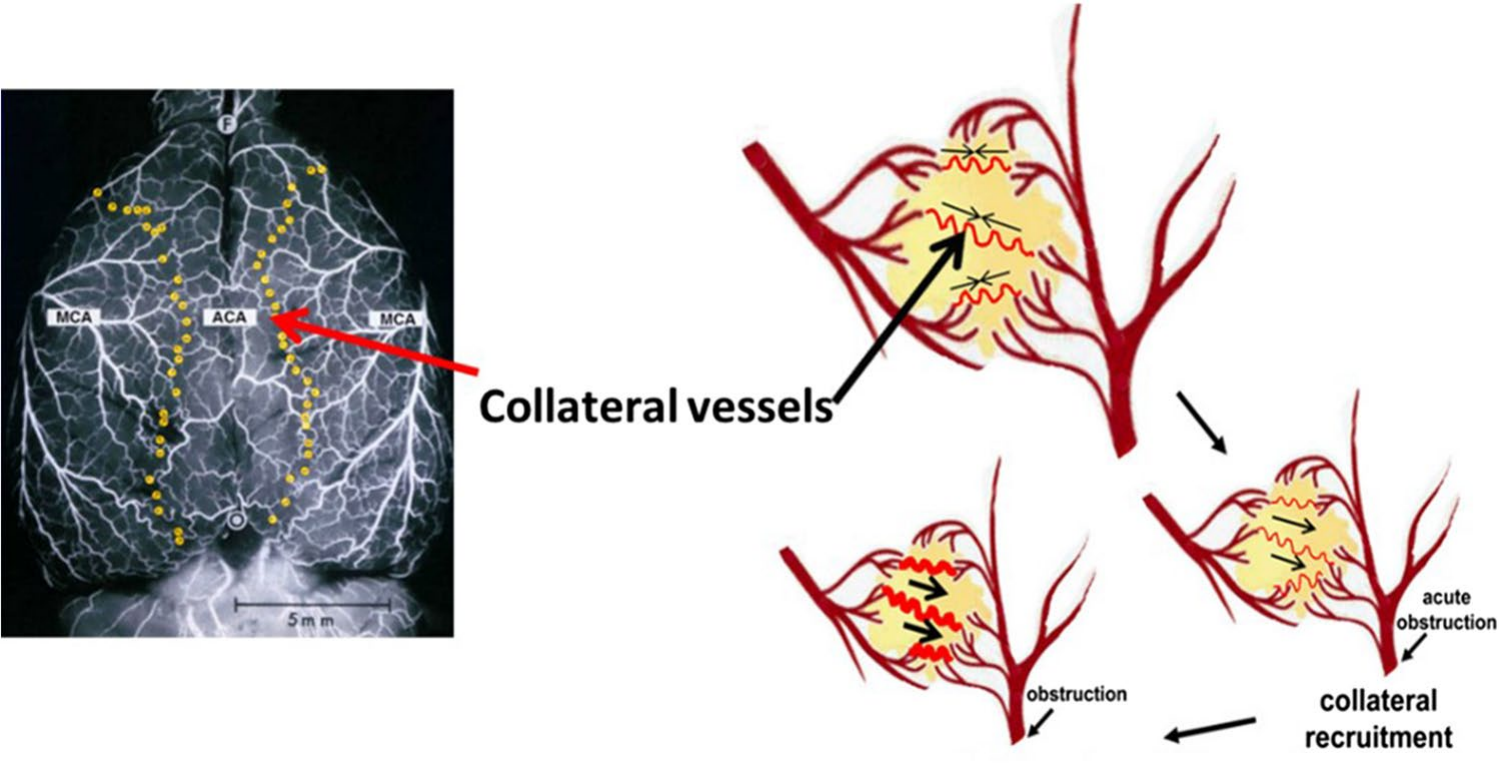
The recruitment of collateral flow. During normal cerebral perfusion, the collateral vessels (LMAs, marked by the yellow beads on the dorsal surface of the rat brain) usually connecting the distal ends of the anterior and middle cerebral arteries have no net flow. The LMAs supply the water-shed territories between major cerebral arteries. However, following the occlusion of a major feeding artery, in the case of MCAO, the intraluminal pressure gradient drives blood from the uncompromised feeding artery towards the ischemic territory to compensate. The collateral vessels further dilate to allow more blood flow over time, if normal blood flow is not re-established. Modified from Coyle and Jokelainen 1982 [31] & Faber et al., 2014 [32]

The cerebral collateral circulation, an anastomosis between the tributaries of major cerebral arteries, [16] helps maintain CBF in the ischemic region following arterial occlusion. Collateral flow is recruited during ischemic stroke where they act to maintain survival of the ischemic penumbra. For example, leptomeningeal anastomoses (LMA’s) are a network of pial arterioles that branch between two major cerebral arteries, most commonly the middle cerebral artery (MCA) and the anterior cerebral artery (ACA). They provide a means of blood flow to the cortical layers of an occluded arterial territory [17]. These vessels open, or are recruited, in response to a variety of direct neural, metabolic, and hemodynamic factors (Fig. 1). Greater collateral recruitment may improve the size and prolong survival time of the ischemic penumbra and reduce reperfusion injury [18]. In fact, much clinical evidence suggests that collateral status is an independent variable in the assessment of ischemic stroke outcomes [19–26].

Further, effective leptomeningeal collateral flow may influence the rate of reperfusion and recanalization following endovascular treatments [22, 23, 27]. Effective collateral flow delivers thrombolytic agents to the clots from more than one side, thereby increasing the efficiency of treatments [28]. Also, the thrombolytic agent is conveyed down to the microvascular level, facilitating the lysis of dislodged clots and ensuring reperfusion in the ischemic territory [29]. Also, collaterals nourish and preserve the integrity of the vasculature within the penumbral territory, thereby preventing endothelial damage and hemorrhagic transformation, commonly observed following endovascular therapies [29]. A recent systematic review and meta-analysis revealed that evidence of good collaterals predicts a beneficial prognostic outcome in patients who received thrombolytic treatment [30]. Hence, improving effective leptomeningeal collateral recruitment during acute ischemic stroke ought to be the objective of current preclinical ischemic research.

### Genetics and Collateral Blood Flow During Ischemic Stroke

Given the existence of considerable morphological variation in the quantity, diameter, and functional recruitment of collateral vessels in humans [16], some patients are predisposed to have effective collateral circulation following stroke while others are not. Two main factors may be responsible for the effective recruitment of collateral vessels following stroke: genetics and absence of stroke comorbidities.

Preclinical evidence from inbred mouse strains have shown that inter-individual variability of collateral extent in the brain depends chiefly on genetic makeup [33–38]. Kao et al. [38] showed the genetic determination of collateral extent following stroke in mice. They used two mouse lines that are genetically similar except for the presence or absence of determinant of collateral extent-1 (Dce1) at a distinct locus on chromosome 7. The inbred mouse strains are CNG-Bc from congenic wild-type BALB/cByJ (Bc) and CNG-B6 from congenic Bc mice with the C57BL/6 J (B6) allele of Dce1 mice. CNG-Bc mice have scanty pial collateral vessels with small diameter while CNG-B6 mice have abundant collateral vessels with large diameter. This was the first study to demonstrate the genetic bases for collateral extent. Using, MRI ADC-deficit volumes, which indicate the amount of tissue injury following MCAO, and perfusion-diffusion mismatch volumes, which indicate the volume of ischemic penumbra, they showed no difference between the mice strains at 1 h following pMCAO. However, at 5 h and beyond, there was a significant difference in ADC-deficit volume and perfusion-diffusion mismatch between the strains, with CNG-B6 having a smaller final infarct volume. Whether this genetic modification of collateral extent in mice is a true representation of clinical observations and the clinical relevance of this genetic determinant of collateral extent is yet to be explored. However, this study indicates that enhanced collateral flow is beneficial to prolong the survival time of the penumbra since the collateral rich mice had significantly greater perfusion mismatch volume and smaller final infarct volume beyond 5 h and at 24 h, respectively, following permanent focal ischemia.

Furthermore, Cristofaro et al. [39] demonstrated that an increased number of native pial collateral vessels alone without a functional recruitment or dilation of these vessels does not increase flow to ischemic territory following MCAO. Notch ligand Delta-like 4 (Dll4) improves arterial differentiation but inhibits the formation of vascular anastomoses. They used genetically modified (Dll4 + / −) mice with loss of the Dll4 function to increase the quantity of native collateral vessels. The quantity of collateral anastomoses did not influence final infarct volume following MCAO in mice, due to loss of anastomotic functional capacity for dilation following focal cerebral ischemia, when compared to wild-type litter mates. In addition, Rab GTPase-effector-binding protein 2 (Rabep2) has been linked to the phenotype of rich collateralization with wider diameter. Rabep2 promotes VEGF-A/VEGFR2 signaling which is responsible for the extent of vascular collateralizations during the embryonic stage of development in mice [40].

Taken together, genetic factors determine the extent and quantity of collateral vessels available for recruitment following stroke. However, genetic disposition alone cannot account for the effective recruitment of collateral vessels following cerebral ischemia. Hence, increased efforts in investigating promising adjunctive CBF-enhancing therapies are urgently needed.

## Potential Adjunctive to Improve Collateral Flow

In addition to the time taken to arrive at the hospital, it takes over 30 min to administer medical treatments for ischemic stroke due to radiological determination of rt-PA and mechanical treatment eligibility [41]. Given the valuable “brain-time” lost prior to standard hospital care, intensified efforts are underway to develop treatment strategies that would improve the functional capacity and flow of collateral vessels in acute ischemic stroke. Certainly, the functional capacity and extent of collateral vessels is more important than the time it takes for treatment to commence as good collateral flow reduces infarct growth and impaired collateral flow accelerates brain lesion development [42–44].

Therefore, enhancing collateral recruitment and flow is critical for maximizing the amount of salvageable brain tissue and improving functional recovery. Here, we consider potential treatment strategies that may enhance the recruitment of collateral flow in the setting of acute ischemic stroke.

- 1. Head position
  Head position lowered with reference to elevated body following stroke has been associated with increased blood flow to the ischemic region [45]. The preclinical randomized trials of Beretta et al. [46] show that during MCAO and following reperfusion, head positioning with rats lying in a backward tilt at an angle 15° showed improved CBF and functional outcome when compared to other strategies to enhance collateral flow such as a 30% increase in mean arterial blood pressure with intravenous phenylephrine, increasing blood volume with polygeline, and carbon dioxide-stimulated cerebrovascular dilation with acetazolamide (100 mg/kg, i.v.). Laser Doppler flowmetry (LDF) was used to monitor cerebral blood flow in this study. In addition, it was revealed in a meta-analysis that stroke patients who lie flat have increased blood flow velocity in the cerebral vessels supplying the ischemic territory compared to patients who lay with their head up at an angle 30° [47]. However, a previously published observational report [48] declares that convincing clinical evidence to support a change of head position in acute stroke is lacking. Also, a report from a randomized clinical trial—The Head Positioning in Acute Stroke Trial (HeadPoST)—observed no difference in functional outcome measures between groups of patients with acute ischemic stroke that had head positioning treatment [49]. A potential contributor to the neutral results of the HeadPoST trial could be the wide entry criteria rather than selecting for patients likely to be collateral dependent. Clinical series in patients with large vessel occlusion and course instability have shown that head positioning can influence outcomes in these uncommon but important patients [50].
  A follow-up report [51] from the same group demonstrated a significant increase in CBF velocity, as quantified by transcranial doppler technology, in patients with anterior ischemic stroke. In addition, Sands et al., [52] showed that change in head position leads to increased blood pressure over time in stroke patients as well as in healthy controls. More importantly, they reported increased CBF in the compromised hemisphere. Perhaps, this strategy may only help improve CBF by increasing blood pressure at the time of stroke. Although some reports [53, 54] indicate that elevated post-stroke blood pressure is beneficial, other reports indicate that raised blood pressure in acute ischemic stroke may impact functional outcome measures due to its association with edema and hemorrhagic transformation [55–57]. However, given that preclinical evidence suggests potential benefits, it stands to reason that a combination of strategies in addition to head positioning might be worth a cursory investigation—especially in patients who are in regions without access to or are ineligible for advanced clinical stroke interventions.
- 2. Neuroprotectants
  Sphingosine-1-phosphate (S1P) belongs to the sphingolipid class of cell membrane-derived lipids and is essential for many cellular processes such as cell growth, survival, motility, angiogenesis, leucocyte migration, and maintaining BBB integrity [58, 59]. S1P is equally expressed in a variety of cells in the brain, including neurons, glial and endothelial cells [59]. In addition, regarding focal cerebral ischemia, S1P agonist (SEW2871 or LASW1238) and the S1P modulator fingolimod (FTY720) have been shown to be neuroprotective in humans [60] and rodents [61–64] through direct neuroprotection, decreased immune cell activation, and decreased microvascular permeability.
  Recently, using mouse descending aorta, in vivo and in vitro, Jung et al. [65] showed that S1P responds to shear stress and modulates flow-mediated signaling in endothelial cells. Also, S1P expression is upregulated in the leptomeningeal arteries under prolonged shear stress and S1P dilated the cortical collateral arteries between the ACA and MCA following the unilateral occlusion of the ICA in rodents [66]. In a mouse model of permanent middle cerebral artery occlusion (pMCAO), Iwasawa et al. [67] showed that S1P agonist, SEW2871, improved collateral flow on day 7 post-MCAO through the phosphorylation of eNOS and reduced infarct volume as well as improved neurological outcome. Given the relevance of S1P to collateral recruitment, further investigations are warranted on the role of S1P during the first critical hours following stroke. It will be informative to test the hypothesis that S1P agonists will maintain the ischemic penumbra within the 4 h of permanent ischemic stroke as well as determine whether reperfusion injury will be ameliorated following the administration of SEW2871.
- 3. Stimulation of the sphenopalatine ganglion
  Intracranial blood flow increases by approximately 40% following the stimulation of the sphenopalatine ganglion (SPG), a structure responsible for the vasodilation of intracranial blood vessels via parasympathetic innervation from the superior salivatory nucleus [68–71].
  The SPG, situated in the pterygopalatine fossa of the skull, supplies parasympathetic innervation to anterior cerebral and meningeal blood vessels [68, 71]. Preclinical studies in rodents, dogs, and primates have established potential benefits of SPG stimulation in the context of stroke. These include augmentation of ipsilateral CBF via arterial vasodilatation, which led to smaller infarct volume and stabilization of the blood-brain barrier (BBB) [68–71]. These vasodilatory effects occur immediately in a frequency-dependent manner and are likely mediated via mechanisms of the neurotransmitters nitric oxide and vasoactive intestinal polypeptide [69, 72, 73].
  Following a 2 h transient (intraluminal filament model of) middle cerebral artery occlusion (MCAO) in Wistar rats, SPG stimulation administered 18 h after MCAO induction significantly reduced brain lesion and improved functional outcome on day 8 but not 28 [73]. This suggests that although delayed, increased CBF through SPG stimulation may promote neuronal survival and improve short term outcome measures when administered after transient MCAO (tMCAO). SPG stimulating electrodes were implanted near the post-ganglionic parasympathetic nerve fibers of the SPG of each rat, 24 h prior to MCAO induction. SPG stimulation, which was applied every 15 min for 3 h each day, entailed 2-min-long pulses (of 2-mA amplitude, 0.5-ms pulse width, and 10-Hz frequency) with a 12-s interval. SPG stimulation was administered for 7 consecutive days after MCAO.
  Similarly, SPG stimulation administered 15 min or 24 h after photothrombotic model of permanent MCAO (pMCAO) induction in adult rats reduced infarct volume and BBB dysfunction [74]. SPG stimulation was delivered in two sets of 60-s stimuli (at 10 Hz, with 12-s interval) for a daily duration of 3 h and 4 consecutive days. These findings were associated with vasodilatation of the cortical arterioles and improved cortical function as measured by laser-Doppler probe and electrocorticography, respectively [74]. The foregoing, delayed SPG stimulation, either in models of reperfusion or permanent ischemia, enhances CBF and ameliorates ischemic lesion while improving functional outcome following sub-acute ischemic stroke.
  To demonstrate the importance of SPG to ischemic stroke outcome, several studies [75–77] reported that disruptions of the SPG or its efferent signals led to substantial decreases in CBF and exacerbated infarct volume in normotensive and hypertensive rats. Corroborative findings have been reported in cynomolgus monkeys, where SPG stimulation increased ipsilateral CBF and decreased arterial vasospasm following subarachnoid hemorrhage [78]. In addition, the Implant for Augmentation of Cerebral Blood Flow Trial in Mild Strokes (ImpACT-24M) trial, a single-arm study, reported that a 4-h daily session of SPG stimulation for 5 continuous days significantly improved cervico-cranial blood flow and decreased hand motor weakness in patients with anterior circulation ischemic stroke [79]. The effects of SPG stimulation were evaluated using two metrics: volumetric blood flow in the ipsilateral common carotid artery assessed by ultrasound, and grasp and pinch strength of the affected hand prior to and post-stimulation. Further, in two multicenter studies, [80, 81] ischemic stroke patients were treated with SPG after up to 24 h of symptom onset. Due to their ineligibility for thrombolytic therapy, these patients did not receive conventional endovascular interventions. However, with respect to 90-day functional outcomes following SPG treatment, statistical significance was attained in a subgroup of patients with cortical infarct when compared to sham controls.
  Hence, SPG stimulation bears no safety concerns but holds significant promises for improving stroke outcomes when administered prior to, and/ or following available reperfusion therapies. Further investigation at the preclinical level should consider aging and comorbidities while clinical trials should expand the number of recruited patients through well-advocated multiple-center studies which will cover areas with multi-ethnic representations. Also, it is pertinent to explore the possible interaction between thrombolytic agents and SPG stimulation, and to better understand the contributions of the different neurotransmitters released by the SPG [82].
- 4. Remote ischemic perconditioning
  Remote ischemic conditioning was first described in 1986 in the context of myocardial infarction [83]. However, the concept has since been experimentally and clinically applied to ischemic stroke. [84–86] Remote conditioning can be administered prior to the induction of ischemic stroke (remote ischemic preconditioning), during ischemic stroke (remote ischemic perconditioning; RIPerC) and following reperfusion (remote ischemic post-conditioning). RIPerC is a more clinically applicable potential (adjunctive) collateral-enhancement therapy since ischemic stroke can only be treated following the onset of neurological symptoms and prior to expert medical diagnosis/ intervention [87]. Hence, RIPerC is an emerging new neuroprotective adjunctive therapy for acute ischemic stroke. It is touted as a therapeutic strategy with the potential to protect the ischemic brain prior to CBF restoration as well as from neuronal reperfusion injury following endovascular treatment [87]. Essentially, RIPerC involves a series of controlled transient cycles of ischemia/reperfusion applied to a non-vital organ such as an unaffected limb, during acute brain ischemia [87, 88]. Although the exact mechanism of remote ischemic conditioning is unclear, experimental and clinical findings suggest it increases CBF in the affected brain region by endogenous humoral mediation [89–93]. Specifically, nitric oxide-mediated dilatation is believed to drive the benefits associated with remote ischemic conditioning since blood levels of nitrate and nitrite were increased in humans and mice following treatment [90]. However, mice deficient in endothelial nitric oxide synthase (eNOS) were found to not benefit from remote ischemic conditioning [90]. Further, mRNA expressions of eNOS in blood vessels as well as plasma levels of nitric oxide were significantly elevated following RIPerC during acute ischemic stroke in mice [90].
  Recently, pre-clinical reports indicate that RIPerC administered by three cycles of bilateral femoral artery occlusion commencing within 1 h of permanent distal middle cerebral occlusion (dMCAO) in normotensive, young adult-[84] as well as in aged male [86], Sprague Dawley rats increases collateral CBF in the ischemic region. Moreover, these studies show the diameter of pial arterioles, as measured by laser speckle contrast imaging (LSCI) and two-photon laser scanning microscopy (TPLSM), was not only increased but maintained in the ischemic region for over 4 h. This suggests that RIPerC prevented the collapse of collateral vessels (as seen in the control cohort) and led to the reduced early infarct volume (after 6 h of permanent dMCAO) in young and aged rats. Interestingly, the dilatation of pial arterioles by RIPerC during acute dMCAO in young and aged Sprague-Dawley rats, was not associated with systemic blood pressure nor vascular flow velocity changes. At any rate, RIPerC administered alone and in combination with rt-PA enhances collateral blood flow and reduces infarct volume in young and aged male rats, ovariectomized female, and middle-aged male mice subjected to dMCAO or embolic models of ischemic stroke [84, 86, 90–92].
  Two prior randomized trials which investigated the potential of RIPerC, as an adjunctive therapy to intravenous rt-PA for acute ischemic stroke, found that treatment administration was feasible in over 240 patients en route to the clinic [85] and in 188 patients following admission [94]. Importantly, while both trials differed in time and duration of administration as well as site of administration (pre-hospital/5 min/ arm [85] and admission/ 40 min/leg [94], respectively), the impact of RIPerC on brain infarct volume in patients administered standard treatment and those who received RIPerC was equivocal. However, a follow-up evaluation demonstrated a higher penumbral tissue volume in patients administered pre-hospital RIPerC [85]. This indicates that RIPerC may extend the life span of the penumbra when administered in a prehospital setting. Since the goal of any ischemic stroke treatment is to preserve and functionally restore the ischemic penumbra, this finding is both exciting and reassuring. However, the efficacy of RIPerC following MT intervention is to be determined, since both studies had stroke patients with large artery occlusion which is the primary indication for lone MT or MT and rt-PA combined treatment. Hence, a proof-of-concept study is warranted in large vessel occlusion stroke patients randomized for pre-hospital RIPerC treatment for up to 40 min followed by MT intervention.
  Certainly, the RIPerC treatment releases humoral factors which mediate the dilation of collateral flow, but also must have various other mechanisms for reducing infarct volume. Previous findings show increased circulating nitrite [95] during RIPerC ameliorates autophagy and blood-brain-barrier dysfunction, [93] thereby reducing hemorrhagic transformation in the ischemic region following experimental murine ischemic stroke. Further studies are warranted to define the mechanism(s) potentiating the benefits of RIPerC during acute ischemic stroke.
- 5. Vasoactive agents
  Various vasodilating agents have been suggested to increase CBF following stroke. The prominent examples are nitric oxide (NO) donors, inhaled NO, soluble guanylate cyclase (sGC) activators and stimulators, rho-kinase inhibitors as well as hemoglobin-based oxygen carriers. These target vasoactive agents will be discussed under two broad sections, NO-dependent and NO-independent vasodilators, as they relate to CBF increases in acute ischemic stroke.
- 6. NO-dependent vasodilators
  NO is a potential therapeutic agent for ischemic stroke and many experimental studies showed that exogenous NO, either via the administration of NO derivative [96, 97] or by direct inhalation [98, 99], increases CBF. An ideal vasodilating agent would selectively increase the diameter and/ or blood flow of the pial collateral blood vessels in the region of hypoperfused brain tissue, to extend the survivability of the salvageable tissue in the ischemic territory.
  Nitric oxide’s role in vasodilation was first recognized in the 1970s and was then referred to as endothelium-derived relaxing factor [100, 101]. About a decade later, it was chemically identified as NO [102] and its application to translational research has taken off successfully. Indeed, in the cascade of molecular events following the onset of brain ischemia, the decreased production and/ or bioavailability of NO was identified along with increased oxidative stress. Early studies which investigated the role of NO in ischemia/reperfusion injury found that the reduced bioavailable NO in the region of ischemia is due to the impedance of endothelial NO synthase activity which is chiefly responsible for NO production in blood vessels [103–105]. Moreover, the increased NO bioavailability is now indicated as a potential therapeutic in the setting of ischemic stroke.
- 7. NO-independent vasodilators
  Newer compounds are in place to circumvent NO-signaling for vasodilation and platelet inhibition by specifically targeting the oxidized and heme-free forms of sGC, which are the abundant subtypes in chronic hypertension [125]. Increased levels of ROS directly oxidize the Fe2+ group of sGC to Fe3+ which makes sGC unresponsive to NO [128, 129]. Also, Fe3+-sGC is easily dissociated from its ferrous group, with continuing oxidation, leading to the formation of heme-free sGC which further decreases NO-signaling. Therefore, the attenuated production of cGMP, as a result of change in the configuration of sGC, would inhibit the physiological response (i.e., vasodilation) brought about by cGMP-dependent protein kinases. Below, we discuss the CBF-enhancing potential of NO-independent vasodilators.

These fundamental research reports clarified the role and capabilities of NO during ischemic stroke and led to seminal reports on pre-clinically administered NO via direct inhalation or through NO donor agents to ameliorate the effect of brain ischemia [96–99].

### Glyceryl Trinitrate

Preclinical publications demonstrate the efficacy and potential benefits of glyceryl trinitrate (GTN) as an adjunctive therapy for ischemic stroke [96, 97].

A recent study investigated the effect of sub-dermal administration of GTN (0.2 mg/h and 0.69–50 µg/h), 2 h after the induction of permanent MCAO in sheep and mice, respectively [97]. They reported no significant difference in infarct volume, CBF, and physiological outcomes following 24 h of ischemic stroke in mice, when compared to vehicle control cohorts. In contrast, the same report showed that GTN significantly prevented the increase in intracranial pressure and reduced infarct volume and cerebral edema without altering mean arterial blood pressure, when compared to vehicle control sheep following 24 h of permanent MCAO. The reported differences between ovine and mouse species in the efficacy of GTN in acute ischemic stroke cannot be readily explained even though there exist cerebro-morphological differences between the species. Firstly, the model and duration of ischemic stroke as well as the route and dosage of GTN administered were comparable between both species. Also, our earlier study [96] showed that GTN reduced infarct volume and improved functional outcome measures when GTN (6.25 and 12.5 µg/µL) was administered intra-arterially.

Nevertheless, all the phase II clinical trials in the UK and elsewhere did not attain the pre-determined desirable outcomes [106–108]. Across three phase II randomized clinical trials: Efficacy of NO in Stroke (ENOS), Rapid Intervention with GTN in Hypertensive stroke Trial (RIGHT), and RIGHT‑2, GTN was demonstrated in acute and subacute stroke patients to lower central and peripheral blood pressure, and improve vascular compliance without compromising CBF and intracranial pressure [106–109]. Lowering systemic blood pressure during the occlusive phase of the ischemic stroke is well documented [110] to be detrimental for outcome measures, as opposed to outcomes in hemorrhagic stroke cases. Since the ENOS, RIGHT, and RIGHT-2 studies included a case mix of both ischemic and hemorrhagic strokes, it is difficult to truly ascertain the safety of GTN in acute ischemic stroke [106–109]. Moreover, the timing of administration of GTN can predict outcome benefits. In the ENOS trial, administering GTN (5 mg) as a transdermal patch within 48 h of symptom onset did not improve measured functional outcomes, 90 days after treatment [106]. On the other hand, administering GTN within 6 h of symptom onset reduced mortality and improved functional outcome without incidence of adverse effects [111]. These results birthed the two other trials (RIGHT-1 and RIGHT-2) which aimed to commence administration of GTN transdermal patch prior to patients’ arrival at the clinic [107, 108]. Unfortunately, the findings of both trials demonstrate that GTN administered within 4 h of symptom onset showed no improvement in functional outcome at 90 days. Hence, although GTN appears safe and has the potential to reduce infarct volume, the functional outcome benefits require further probe for efficacy.

### Inhaled NO

Terpolilli et al. [98] showed that inhaled nitric oxide (iNO) selectively dilates blood vessels in the ischemic region following stroke without affecting normal tissue perfusion in pig and mice models. Also, iNO enhanced collateral recruitment following neonatal hypoperfusion in mice models [99]. A year later, the study by Li et al. [112] showed that the benefits of iNO in acute ischemic stroke are duration and dose dependent. Hence, 60 ppm for 4 h had an optimal and beneficial outcome in mice. While these preclinical findings emanated from the study of normotensive animals, we conducted a similar study with the aim of investigating the effect of iNO on cortical collateral perfusion following pMCAO in spontaneously hypertensive stroke-prone rats (SHRSP). We found no effect of iNO on cortical collateral blood flow in SHRSP [113]. This may be due to impaired NO-signaling occasioned by chronic hypertension as observed in hypertensive rats [114, 115] and humans [116–118].

Specifically, hypertension is associated with decreased expression of sGC and an attenuated vascular response following the administration of NO-donors [115, 119–125].

Moreover, oxidative stress is particularly increased in the brain of SHRSPs [126, 127]. A further rise in oxidative stress levels following focal cerebral ischemia in the SHRSPs contributes to the pathophysiology of ischemia. Hence, the therapeutic potential of iNO in ischemic stroke models with hypertension is limited due to diminished NO signaling and raised oxidative stress levels which will result in a neutral effect on CBF.

In light of the aforementioned, it is conceivable that NO signaling is compromised due to exaggerated oxidative stress following focal cerebral ischemia in chronic hypertension. Hence, increasing the bioavailability of NO, by inhalation or the administration of NO donor, may not translate to improvements in the recruitment of collateral blood vessels following stroke in these models—and by extension in patients with a history of chronic hypertension.

### Soluble Guanylate Cyclase Activators

Stroke comorbidities such as hypertension and diabetes elevate oxidative stress, which leads to the oxidation of Fe^2+^-sGC [130]. Therefore, sGC activators which bind to the Fe^3+^- and heme-free sGC, rather than *sGC-stimulating* compounds that target the reduced (Fe^2+^) form of sGC, are ideal for enhancing collateral flow following focal ischemic stroke in comorbid models [131]. These NO-independent and heme-independent sGC activators include BAY 58–2667 (cinaciguat) and BAY 60–2770 from Bayer Pharmaceuticals, and S-3448 and HMR-1766 (ataciguat) from Sanofi Aventis [132, 133]. In a series of mice studies, Langhauser et al. [134] showed that the heme-free sGC activators BAY58-2667 or BAY60-2770 (at 30 μg/kg and 10 μg/kg, respectively) increased neuroprotection following 24 h of pMCAO and tMCAO in mice. Also, using laser doppler flowmetry they reported an increased CBF in treated mice following pMCAO and tMCAO, when compared to control. Although they demonstrated the capacity of sGC activators to dilate cerebral blood vessels, which resulted in small infarct volumes following stroke, they did not specifically investigate the impact of these compounds either on collateral flow or in the presence of stroke comorbidities. Further, the potential of sGC activators to increase CBF and/ or collateral flow is yet investigated in aged and comorbid preclinical models.

### Sanguinate

Sanguinate, a non-NO-dependent selective vasodilator was reported by Cipolla and colleagues [135] to significantly increase CBF during 90 min MCAO and following reperfusion in young adult spontaneously hypertensive rats (SHRs). Similarly, during pMCAO in normotensive Wistar rats, 10 ml/kg of PEG-COHb resulted in increased perfusion in the “penumbral region” but not in the ischemic core and without altering mean arterial blood pressure [136]. The exact mechanism by which Sanguinate brings about enhanced collateral flow is unclear. As a PEG-COHb gas transfer agent, Sanguinate can deliver CO, [137] which is known to stimulate the action of sGC. However, the sGC stimulation theory may not apply to Sanguinate in ischemic stroke, because CO can only bind to Fe ^2+^-sGC, which are less abundant in essential hypertension. Nevertheless, by the selective release of O_2_ to ischemic brain tissue with low arterial oxygen tension and saturation, Sanguinate is potentially able to maintain the penumbra during occlusion as well as preserve neuronal integrity following reperfusion. In summary, it is clear that the potent vasodilatory capacity of Sanguinate is independent of NO-signaling and the exact mechanism by which Sanguinate dilates cerebral vessels following focal ischemic stroke in hypertensive and normotensive models is subject to further investigations.

It is important to determine the evolution of penumbra as well as the dynamic recruitment of collateral flow following the administration of these NO-independent heme-independent sGC activators as well as Sanguinate in models of stroke comorbidities.

### Fasudil

Rho-associated protein kinase (ROCK) consists of two cellular subtypes, ROCK1 and ROCK2, which are both expressed in the brain and regulate basal tone of peripheral and cerebral blood vessels [138] by inhibition of myosin light chain phosphatase (MLCP). Myosin light chain (MLC) phosphorylation regulates vascular smooth muscle cell contractility, and many vasoactive mediators (such as angiotensin II, endothelin-1, and platelet-derived growth factor (PDGF)) mediate vasoconstriction via ROCK interaction [139]. Activation of ROCK decreases NO production through the inhibition of eNOS [140]. Importantly, ROCK function and activity is enhanced in cerebral and non-cerebral blood vessels in hypertension [141, 142] and following cerebral ischemia [143]. This suggests that in the presence of hypertension and following stroke, enhanced ROCK activity may contribute to poorer collateral flow and poorer recovery of perfusion following recanalization.

Fasudil, a non-specific inhibitor of ROCK1 and 2, inhibits phosphorylation of MLCK, increases eNOS expression, and causes vasodilatation of cerebral blood vessels [143]. In vivo studies show that inhibition of ROCK by Fasudil improves the perfusion deficit following experimental stroke, [144] an effect that may be mediated through an augmentation of collateral supply [145]. Narrowing of cerebral blood vessels was attenuated in streptozotocin-induced diabetic rats treated with Fasudil and infarct volume following experimental stroke was reduced in diabetic rats chronically treated with Fasudil [146]. Also, Yagita and colleagues [147] showed increased eNOS phosphorylation in the brains of non-stroke rats after Fasudil injection, and following cerebral ischemia, Fasudil treatment reduced infarct volume, an effect associated with improved activity of eNOS.

Moreover, Lee and colleagues [148] carried out a comprehensive series of experiments using the ROCK2 selective inhibitor (KD025 formerly SLx-2119) in a mouse model of focal cerebral ischemia (permanent and transient) and demonstrated a dose-dependent reduction in infarct volume—an effect that may be partially attributed to enhanced collateral flow [148]. In this series of experiments, the authors demonstrated that pre-treatment with the ROCK2 inhibitor reduced the perfusion deficit following distal MCAO, suggesting that ROCK2 inhibition modulates CBF during occlusion. In addition, the protective effects of KD025 on reducing infarct volume following transient MCAO were still observed when administered 1 and 3 h following stroke, and protective effects on outcome were still maintained when given to co-morbid animals (aged and diabetic mice) [148]. Constriction of cerebral arteries and myogenic tone of parenchymal arterioles were recently shown to be ROCK2 dependent and the study authors suggest that ROCK2 is a major regulator of vascular tone in muscular arteries and resistance vessels [149].

To date, the studies investigating the effect of ROCK inhibition on CBF usually administer treatment prior to stroke induction. Since stroke can only be clinically managed following symptom onset, potential stroke therapies are only translatable if administered following ischemic stroke. Given the promising effect of Fasudil on CBF enhancement, more studies are urgently required to investigate the CBF impact of Fasudil following MCAO induction. Indeed, one study reported that low-dose Fasudil did not increase CBF during MCAO in hypertensive rats [150]. While this study deliberately used low dose of Fasudil due to potential decrease in mean arterial blood pressure, we reason that administering previously established effective dose of Fasudil may be beneficial for CBF enhancement in permanent MCAO models. In addition, a recent systematic review and meta-analysis of the efficacy of ROCK inhibitors in animal models of stroke demonstrated that ROCK inhibitors appear to be effective. However, many of the studies included in the meta-analysis were determined to be of low methodological quality based on factors influencing bias such as lack of blinding and randomization as well as the lack of animal models displaying co-morbidities [151]. The authors conclude that further high-quality studies are needed in order to determine the potential efficacy of ROCK inhibitors for stroke.

## Future Direction and Consideration for Clinical Relevance

Overall, the viability of cerebral collateral flow following ischemic stroke indicates the feasibility of enhancing collateral flow as a therapeutic strategy to preserve penumbral tissue before and/ or following clinical intervention with thrombolysis and thrombectomy. While CBF enhancement is essential in acute stroke care, increased oxygen delivery to the penumbra is equally important. The pre-hospital administration of specific brain oxygen-increasing therapeutics such as normobaric hyperoxia [152] in combination with CBF enhancing strategies should be considered to increase the life span of the penumbra and mitigate reperfusion injuries.

An ideal pre-hospital therapeutic strategy to enhance collateral circulation should satisfy the following ideal conditions: ease of administration, pharmacological safety, and rapid beneficial effect [7, 153]. Nevertheless, to document the safety of potential therapy in hemorrhagic stroke and mimics, a favorable controlled study with the aim to administer treatment in the primary care center and continued in the ambulance while transferring the patient to an expert endovascular center should be considered. From the foregoing discussion, the promising strategies to enhance collateral flow following ischemic stroke include heme-free sGC activators, Sanguinate, remote ischemic perconditioning, Fasudil, S1P agonists, and stimulation of sphenopalatine ganglion (see Table 1 for a summary of outcome measures).

**Table 1:** Summary of articles included in the review

Author and year	Potential CBF enhancer	Outcomes	Notes/confounders	Species/strain	Sex and age	Model/time of stroke
Beretta et al. 2017 [46]	Head position: 15° downward tilt	15% and 25% CBF increase on the lateral and medial MCA territories	Did not address effect of single collateral therapeutics on biochemical pathways in neural cells	Wistar rat	Adult male	90 min transient MCAO, 24 h survival
Aries et al. 2013 [48]	Head position	No difference in CBF velocity and outcomes for postural positioning of patients	Patients included in the study had mild/moderate strokes. Cannot generalize findings to severe strokes	Human	Adult patients: age 62 + / − 15	MCA territory stroke
Anderson et al., 2017 [49]	Head position: elevated 30°	No significant difference in disability outcomes between groups	Most patients had assigned head position implemented after the time window for thrombolytics/endovascular therapy had passed. Earlier head position may have led to different results	Human	Adult patients	Acute stroke diagnosis
Olavarria et al. 2018 [51]	Head position: lying flat	CBF velocity increase in flat lying position compared to upright head position. Improvement in CBF velocity did not translate to better clinical outcomes	Cluster randomized phase 2b trial. Most participants were from a single center, imbalances in clinical characteristics of sample size	Human	Adult patients	Anterior circulation ischemic stroke
Sands et al. 2020 [52]	Head Position: gradual upward tilt from 0 to 30°	CBF velocity increased in the ipsilateral hemisphere. MABP increase over time drove CBF velocity only in the contralateral hemisphere	Small sample size (*n* = 15)	Human	Adult patients: Age 57 + / − 16	Acute ischemic stroke within 24 h of symptoms: total anterior, partial anterior, posterior, and lacunar strokes
Sare et al. 2009 [53]	High blood pressure (BP)	High systolic BP and variability in SBP are associated with poorer functional outcomes	performed observational analysis on randomized controlled trials: inclusion criteria are uncertain; lacks data on stroke subtype (ant. vs. pos.)	Human	Adult patients	Hyperacute ischemic stroke (< 8 h)
Aslanyan et al. 2003 [54]	BP	Elevated average MAP in acute ischemic stroke is associated with poorer outcome/mortality	Cannot prove causality: whether BP changes led to poor outcomes or if they occurred secondary to another cause. Progression of stroke could have preceded BP measurements	Human	Adult patients	Ischemic stroke
Stead et al. 2005 [55]	BP	Low BP and high BP resulted in higher mortality risk compared to normotensive range	Observational study; lack of serial blood pressure measurements; lack of causality that abnormal BP causes mortality	Human	Adult patients	Stroke within 24 h of symptom onset
Ishitsuka et al. 2014 [57]	BP	High BP is associated with poorer clinical outcome and lower probability of neurologic recovery	BP was not measured continuously, instead several measurements were taken; most patients had minor strokes	Human	Adult patients	Stroke within 24 h of symptom onset
Fu et al. 2014 [60]	Neuroprotectants: fingolimod	Lowered circulating lymphocytes, milder neuro deficits, decreased microvascular permeability, smaller lesion size	Small sample size (*n* = 22), nonrandomized duration of enrollment, heterogeneous lesion size	Human	Adult patients	Anterior cerebral circulation occlusion; stroke onset > 4.5 h
Hasegawa et al. 2010 [61]	Neuroprotectants: FTY720 (sphingosine 1-phosphate R agonist)	Significantly reduced infarct volume and neuronal death (deactivation of caspase-3); improved neurological score		Sprague–Dawley rats	Male	120-min transient MCAO, 24-h or 72-h survival
Czech et al. 2009 [62]	Neuroprotectants: FTY720 (sphingosine 1-phosphate R agonist)	Reduced lesion size, improved neurologic outcome, decreased infiltrating neutrophils, reduced apoptotic cell death in the lesion	Reduced infiltration of immune cells into ischemic lesion may simply be due to low peripheral WBC counts	C57BL6/J mice	10-week, male	90-min transient MCAO, 24-h survival
Wei et al. 2011 [63]	Neuroprotectants: FTY720 (sphingosine 1-phosphate receptor agonist [S1PR1])	Reduced infarct size, neurologic deficit, edema, number of dying cells in the core and periinfarct area. Decreased inflammation (active neutrophils, microglia/macrophages, ICAM-1), better behavioral tests	Both decreased neutrophil activation and direction action on endothelial S1P receptors may explain the decreased edema. FTY720 may also limit levels cytotoxic agents (peripheral lymphopenia) rather than direct neuroprotection. May also reduce inflammation through direct effects on endothelium (decreased ICAM-1). Only investigated in model within 24 h due to high mortality rate after that time	C57BL/6 mice	Male	90-min transient MCAO, 15-day survival
Brait et al. 2016 [64]	Neuroprotectants: S1PR1 (fingolimod or LASW1238)	Selective S1P1 agonist LASW1238 better reduced infarct volume than fingolimod	Study suggests that lymphopenia is important in the beneficial effect	C57BL/6 J mice	Adult male	45-min transient MCAO, 24-h survival
Ichijo et al. 2015 [66]	Neuroprotectants: S1PR1 agonist, SEW2871	Significant increase in CBF and diameter of leptomeningeal collateral vessels which resulted in a significantly smaller infarct volume and better functional recovery	A previous study revealed that the neuroprotective effects of SEW2871 and nonselective S1PR1 agonist FTY720 occurred through apoptosis prevention. This study provides evidence that the enhanced collateral recruitment of leptomeningeal arteries is in response to S1PR1-selective agonist	CB7BI/6 mice	Male, 10–14 weeks	Unilateral common carotid artery occlusion for 14 days and subsequent permanent MCAO (pMCAO), 7-day survival
Iwasawa et al. 2018 [67]	Neuroprotectants: S1P agonist, SEW2871	Improved leptomeningeal collateral flow through phosphorylation of eNOS; reduced infarct volume and neurologic outcome		BALB/c mice	Male mice	pMCAO, 7-day survival
Suzuki et al. 1991 [68]	SPG stimulation	Up to 6.3% dilation of pial arteries		Sprague–Dawley rats		
Ayajiki et al. 2005 [69]	SPG Stimulation	> 30% CBF increase in the rat	NO released from parasympathetic nerves and neuropeptides from sensory nerves may be responsible for CBF increase	Wistar rat	Adult male	
Levi et al. 2012 [74]	SPG stimulation	> 400% CBF increase as well as > 10% vasodilation of cortical arterioles; BBB opening, attenuated lesion volume		Sprague–Dawley rats	Adult male	Rose bengal photothrombosis
Kano et al. 1991 [75]	Parasympathetic n	Parasympathetic denervation increases infarct volume		Long Evans rats	Adult male	45 min MCAO combined with bilateral common carotid artery occlusion (CCAO)
Koketsu et al.1992 [76]	SPG stimulation	Sectioning the parasympathetic innervation to the circle of Willis increases infarction volume by 30%		SHRs	Male, young adults	Tandem MCAO/CCAO
Diansan et al. 2010 [77]	SPG stimulation	NOS contained nerves from the SPG are important in decreasing infarction volume	Nerves originating from the SPG also release neurotransmitters besides NO, such as neuropeptide Y, VIP, which may also play a role in ischemia	Sprague–Dawley rats	Male	pMCAO, 24-h survival
Saver et al. 2019 [79]	SPG stimulation	4 h daily session of SPG stimulation for 5 days improved blood flow, vessel diameter, flow velocity, and decreased hand motor weakness	Not a randomized, controlled trial; cervico-cranial blood flow and hand motor strength were assessed in nonblinded manner and only ipsilaterally; quantitative CCA is known to overestimate flow volume	Humans	Both, median age 66	Anterior circulation ischemic stroke
Ma et al. 2017 [84]	Remote ischemic perconditioning	Up to 20% post-MCAO diameter increase as well as reduction in infarct volume in RIPerC rats compared to control	Diameter measures are relative to post-MCAO but prior to treatment, and no measurements were taken of how dilated each vessel was compared to baseline: statistical relationship may be even stronger than presented	Sprague Dawley rats	Male, 2–5 months old	Distal MCAO, 6-h non-recovery
Hougaard et al. 2014 [85]	Remote ischemic perconditioning	MRI studies showed no significant difference in penumbral salvage, final infarct size, and infarct growth. However, there was an overall reduction in risk of infarction for RIPerC tissue	Follow-up group only included MRI-proven stroke and not randomized stroke mimic or TIA; randomization in the study was unequal due to a procedural error which might affect the data	Humans	Male and female adults	TIA, acute ischemic stroke, or hemorrhagic stroke
Ma et al. 2020 [86]	Remote ischemic perconditioning	RIPerC enhanced collateral flow by preventing narrowing of pial arterioles during ischemia, leading to decreased tissue damage	Study used urethane anesthetized animals and cranial windows were used for stable vascular imaging. Use of un-anesthetized animals and a thinned skull preparation can reduce confounds due to skull removal and replacement with a glass window	Sprague Dawley rats	Aged (16–18 months)	Distal MCAO, 6-h non-recovery
Hoda et al. 2012 [90]	Remote ischemic perconditioning	90% CBF increase and 50% infarct volume reduction with combined treatment of RIPerC and tPA when compared to individual treatment or control		C57BL/6 J Mice	20–21 weeks old, male	embolic MCAO, 48-h survival
Hoda et al. 2014 [91]	Remote ischemic perconditioning	> 75% increase in CBF and 40% decrease in infarct volume with combined treatment of RIPerC and minocycline when compared to individual treatment or control		C57BL/6 J mice	Mid-age (12 months) male	embolic MCAO, 48-h survival
Hoda et al. 2014 [92]	Remote ischemic perconditioning	> 70% CBF increase, reduction in infarct volume (> 40%), hemorrhagic transformation (> 20%) and edema (> 5%) after RIPerC treatment, with and without tPA		C57BL/6 J mice	20–21 weeks old, female	embolic MCAO after 7–9 weeks of ovariectomy, 24-h survival
Ren et al. 2015 [93]	Remote ischemic perconditioning	Reduced BBB breakdown, edema, infarct volume, and improved neurologic outcomes		Sprague Dawley rats	Adult male	90-min transient MCAO, 48-h survival
Maniskas et al. 2018 [96]	Glyceryl trinitrate	Acute post-stroke intra-arterial GTN did not change vessel dilation after 15 min, but significantly reduce infarct volume		C57/BL6/J mice	16 weeks old, male	60-min MCAO, 7-day survival
Sorby-Adams et al. 2021 [97]	Glyceryl trinitrate	1. Sheep: GTN administration was associated with decreased ICP, infarct volume, and cerebral edema. No change in BP or cerebral perfusion pressure 2. Mice: No improvement in infarct volume or neurologic score	During ovine data collection, the GTN-treated sheep unexpectedly changed locations and breathed pure oxygen rather than room air, which causes vasoconstriction and reduced CBF. Lack of randomization in the ovine studies	Merino sheep C57BL/6 mice	Female sheep, 18–24 months Male mice, 10–12 weeks old	MCAO
ENOS Trial Investigators 2015 [106]	Glyceryl trinitrate	Transdermal glyceryl trinitrate lowered BP but did not improve functional outcomes	Glyceryl trinitrate was given in a single-blind manner. Some patients receiving glyceryl trinitrate did not receive it for the treatment period of 4 days. The first dose of transdermal glyceryl trinitrate had a smaller effect than was seen in pilot trials (reason unknown). The trial started in 2001, but 79% of patients were enrolled in 2008, who would have benefitted from modern treatments such as thrombolytics and statins	Human		Acute ischemic or hemorrhagic stroke and raised systolic BP
Ankolekar et al. 2013 [107]	Glyceryl trinitrate	GTN reduces SBP and is safe in treatment of ultra-acute stroke. Effect of GTN on functional outcome still needs to be studied	Small sample size	Humans		Ultra-acute stroke (< 4 h) and SBP > 140 mmHg
RIGHT-2 Investigators 2019 [108]	Glyceryl trinitrate	Prehospital (paramedic) administration of transdermal GTN does not improve neurologic outcome	Single blind design; many patients might have received incomplete treatment; had to increase sample size unexpectedly due to high mimic rate; broad inclusion criteria included patients that would not normally have been in the trial	Humans	Adults	Stroke within 4 h of onset, SBP > 120
Rashid et al. 2003 [109]	Glyceryl trinitrate	GTN lowered MAP on day 1 by 5.3%-6.7%. Increasing the dose from 5 to 10 mg on day 5 reduced the BP by 11.4%. GTN reduced peripheral blood pressure, intracranial blood pressure. It did not affect MCA blood velocity or pulsatility index, suggesting CBF was unchanged	Open label rather than placebo controlled (observer bias); transcranial doppler and pulsatility index are indirect measures of CBF; small size of trial	Humans		Acute ischemic or hemorrhagic stroke (< 72 h)
Woodhouse et al. 2015 [111]	Glyceryl trinitrate	Transdermal GTN improved outcome and reduced deaths within 6 h of stroke onset	Small number of patients (type I error); minor imbalances—patients in the GTN group had more mild stroke and fewer cortical stroke syndromes	Humans	Mean age 69.9	Stroke < 6-h onset
Terpolili et al. 2012 [98]	Inhaled NO	Dilation of pial arterioles by 22% in ischemic conditions and 40% increase in CBF in ischemic penumbra; no effect under normal conditions		Mice, Merino sheep	Mice—male, 6–10 weeks old; sheep—female, adult	pMCAO or 45-min transient MCAO, up to 72-h survival
Charriaut-Marlangue et al. 2012 [99]	Inhaled NO	20 ppm iNO increased blood flow velocities by 9.9 cm s^−1^ and reduced lesion volumes by 43%	iNO appears to be either neuroprotective or neurotoxic depending on dosage as well as temporal and spatial factors	Wistar rats	Neonates	MCAO + bilateral common carotid artery occlusion
Li et al. 2013 [112]	Inhaled NO	60 ppm iNO mean infarct size was 14%; air control mean infarct size was 19.3%		Swiss Webster mice	Male	tandem MCAO/50-min transient CCAO
Biose et al. 2020 [113]	Inhaled NO	No significant difference in cortical perfusion between 60-ppm iNO and vehicle-treated rats	No assessment of infarct volume	Spontaneously hypertensive stroke-prone rats	Male	pMCAO, 4-h non-recovery
Langhauser et al. 2018 [134]	sGC activators: BAY60-2770	> 250% CBF increase was observed 4 h after reperfusion in BAY60-2770 (10 μg/kg, i.v.) treated group, but not in pMCAO group		C57BL/6 Mice	8 weeks old or 1 year old, male and female	pMCAO or 60-min transient MCAO, 1-h or 4-h survival respectively
Cipolla et al. 2018 [135]	sGC activators: sanguinate	29% CBF increase and 10% reduction in infarct volume were observed with Sanguinate treatment compared to the vehicle group		Spontaneously hypertensive rats	16–18 weeks old, male	30- or 90-min transient MCAO, 2-h post-reperfusion survival
Zhang et al. 2012 [136]	sGC activators: PEG-COHb	During pMCAO in normotensive Wistar rats, 10 ml/kg of PEG-COHb resulted in increased perfusion in the “penumbral region” but not in the ischemic core and without altering mean arterial blood pressure		Wistar rats	Young adult, male	120-min transient MCAO, 3-day survival
Rikitake et al. 2005 [143]	Rock inhibitor: Fasudil	10 mg/kg IP, increased regional CBF; stimulated 1.5- and 2.3-fold increase in eNOS activity and NO production, respectively. Also, Fasudil reduced infarct size by 33%, and improved neurologic deficit score by 37%	Fasudil was treated 2 days prior to brain ischemia	Mice with C57BL/6 and SV129 mixed background	Not given	MCAO
Shin et al. 2014 [144]	ROCK inhibitor: Fasudil	10 mg/kg IP, decreased both baseline arterial blood pressure and cerebrovascular resistance (CVR) by 15%, and significantly improved tissue perfusion during dMCAO		ApoE KO mice	Male	distal MCAO
Shin et al. 2007 [145]	ROCK inhibitor: Fasudil	10 mg/kg IP, improved CBF in ischemic core and penumbra regions which resulted in reduced infarct area when compared to control		Rats, C57BL/6 J mice		Distal MCAO
Mu et al. 2016 [146]	ROCK inhibitor: Fasudil	10 mg/kg IV, > 20% increased vessel density and reduced infarct volume. Also, Fasudil ameliorated L-NAME-induced regional constriction		Sprague Dawley rats	Male, Young adult	90-min transient MCAO
Yagita et al. 2013 [147]	ROCK inhibitor: Fasudil	10 mg/kg IP, decreased infarct volume and improved eNOS formation	Fasudil was administered immediately after MCAO induction	Wistar rats	Male, adult	80-min transient MCAO, up to 7-day survival
Lee et al. 2014 [148]	ROCK inhibitor: KD025 or SLx-2119	200 mg/kg IP, improved perfusion deficit and reduced infarct volume when compared to vehicle control	KD025 was administered prior to and following MCAO induction	C57BL/6 mice, Type 2 diabetic mice	Male and female, 2–3 months old, 12 months old	MCAO
Chan et al. 2017 [150]	Fasudil	Minimal increase in CBF during and after MCAO when compared to control. Also, no difference in infarct volume	Low-dose Fasudil (0.1 mg/kg i.v.) was administered	Spontaneously hypertensive rats	Male, 16–19 weeks old	2-h transient MCAO, 2-h post-MCAO survival
Keum et al. 2009 [34]	Genetic variation	A major locus (Civq1) on chromosome 7 accounts for 50% of the variation in collateral status improved brain infarct in mice		Mice	12 weeks old	MCAO
Zhang et al. 2010 [35]	Genetic variation	Reduced infarct volume varies by genetic influence on the functional capacity of collateral vessels		C57BLKS/J(BLKS), FVB/NJ(FVB/N), CBA/J(CBA), DBA/2 J (DBA/2), NOD/ShiLtJ(NOD), SJL/J(SJL), 129S1/SvImJ (129S1), C57BL/6 J(B6), NZW/LacJ(NZW), KK/HlJ(KK), C3H/HeJ(C3H), A/J(A), AKR/J(AKR), BALB/cJ(BALB), and SWR/J(SWR) mice	Male, 10–12 weeks old	MCAO
Wang et al. 2010 [36]	Genetic variation	Collateral vessel quantity, diameter, and conductance are specifically influenced by a 172-kb region containing 9 candidate genes on chromosome 7 QTL	N/a	F1 progeny obtained from reciprocal matings of C57BL/6 J (B6) and BALB/cByJ (BALB/c, Bc) were mated to produce an F2 population	N/a	MCAO
Kao et al. 2017 [38]	Genetic variation	Congenic Bc mice with the determinant of collateral extent-1 (Dce1) allele as rich collateral number, diameter, and conductance which resulted in improved perfusion and infarct volume at 5 h of MCAO when compared to the collateral-poor mice (BALB/cByJ (Bc))		BALB/cByJ (Bc) and CNG-B6 mice	Male, 3–5 months old	pMCAO
Cristofaro et al. 2013 [39]	Genetic variation	Absence/loss of The Notch ligand Delta-like 4 (Dll4) improves collateral branching but fails to improve collateral functional capacity, hence Dll4 + / − mice have poor stroke which is comparable to WT mice		Dll4 + / − or WT mice	Female, 8 weeks old, 12 weeks old	Distal MCAO

Advanced age is associated with reduced collateral quantity, lumina diameter, and functional recruitment [154]. Additionally, stroke patients often present with a combination of stroke comorbidities such as chronic hypertension, chronic and acute hyperglycemia, hyperlipidemia, and obesity). Most studies using preclinical models of stroke often do not consider these comorbidities and are at best studied in isolation rather than in combination. Therefore, for relevant translational value, it is pertinent to consider advanced age along with either the presence of acute post-stroke hyperglycemia, chronic hyperglycemia, or chronic hypertension when establishing protocols and rodent models to investigate potential collateral flow-enhancing strategies [155]. Also, since female animal models respond differently to ischemic insults due to higher estrogen circulation, it is important to consider the benefits of testing potential therapy in female and male models of ischemic stroke in the same experimental protocol [156–160].

Investigating CBF changes often require the administration of general anesthesia to preclinical models. However, some anesthesia can lower blood pressure and induce cerebral dilatation (e.g., isoflurane [161–163]) as well as decreased CBF (Urethane [164]). Following stroke, cerebral autoregulation is impaired, and alteration of mean arterial blood pressure will impact CBF [161, 165]. Hence, it is instructive to use anesthetic cocktails/regimens which have minimal effects on central and peripheral circulation. Also, CBF studies can be measured in awake immobilized rodents, which often requires that the cohort of animals are only subjected to non-survival procedures. Appropriate anesthetic regimens for CBF studies in rodents are reviewed elsewhere [166, 167]. Also, the model of MCAO is important for successfully measuring CBF in acute ischemic stroke. An ideal model will be a permanent MCAO model, which should either be embolic MCAO, intraluminal filament/suture model, and photothrombotic models. These models will appropriately measure any increases in CBF feeding from collateral blood vessel dilatation/ recruitment. The benefits and limitation of these models are described elsewhere [168, 169]. The majority of the preclinical models cited in Table 1 used the intraluminal filament model of the MCAO. Future screening studies should endeavor to add one more MCAO model such as distal MCAO or embolic MCAO in order to increase the translational significance of experimental findings.

Finally, some comorbidities such as chronic essential hypertension and acute post-stroke hyperglycemia induce a rapid evolution of the penumbra and expansion of the infarct volume in rodent models of ischemic stroke [170–172]. So, ideal collateral flow enhancing strategies would demonstrate the potential to rapidly ameliorate the impaired collateral recruitment within the first 2 h from ischemia onset.

## Conclusions

**Fig. 2:**
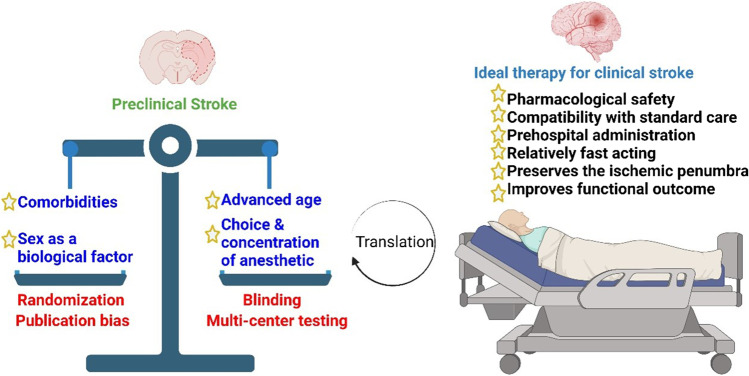
Improved translational impact of preclinical studies. All experimental stroke studies designed to improve stroke outcomes should be subjected to the proverbial scale of the STAIR and ARRIVE guidelines. Comorbidities, sex as a biological factor, advanced age, and choice and concentration of anesthetic agents should constantly be refined to identify the ideal therapeutic strategy for clinical stroke

We have enumerated several promising avenues to improve cerebral blood flow in acute ischemic stroke. Most stroke patients often present with more than one comorbidity (such as hypertension, hyperglycemia, hyperlipidemia, obesity, etc.), yet more studies continue to use young and otherwise healthy preclinical models to investigate potential strategies to improve CBF. While we realize that young animals can serve a vital role for the screening of potential therapies, we reason that the improvement of translational impacts of experimental ischemic stroke efforts will be best served when advanced age with chronic hypertension and other acute/ chronic comorbidities of stroke are incorporated in the testing of potential adjunctive therapies to enhance CBF (Fig. 2).

## Abbreviations

ACA: Anterior cerebral artery
BBB: Blood-brain barrier
BP: Blood pressure
CBF: Cerebral blood flow
CCAO: Common carotid artery occlusion
CVR: Cerebrovascular resistance
Dce1: Determinant of collateral extent-1
Dll4: Delta-like 4
dMCAO: Distal middle cerebral artery occlusion
eNOS: Endothelial nitric oxide synthase
ENOS: Efficacy of NO in Stroke
GTN: Glyceryl trinitrate
HeadPoST: The Head Positioning in Acute Stroke Trial
ICA: Internal carotid artery
ICAM-1: Intercellular adhesion molecule 1
ICP: Intracranial pressure
ImpACT-24M: Implant for Augmentation of Cerebral Blood Flow Trial in Mild Strokes
iNO: Inhaled nitric oxide
LDF: Laser Doppler flowmetry
LMA: Leptomeningeal anastomoses
LSCI: Laser speckle contrast imaging
MAP: Mean arterial pressure
MCA: Middle cerebral artery
MCAO: Middle cerebral artery occlusion
MLC: Myosin light chain
MLCP: Myosin light chain phosphatase
MRI ADC-deficit: Magnetic resonance imaging apparent diffusion coefficient-deficit
MT: Mechanical thrombectomy
NO: Nitric oxide
PDGF: Platelet-derived growth factor
pMCAO: Permanent middle cerebral artery occlusion
Rabep2: Rab GTPase-effector-binding protein 2
RIGHT: Rapid Intervention with GTN in Hypertensive Stroke
RIPerC: Remote ischemic perconditioning
ROCK: Rho-associated protein kinase
rt-PA: Recombinant tissue plasminogen activator
S1P: Sphingosine-1-phosphate
SBP: Systolic blood pressure
sGC: Soluble guanylate cyclase
SHRs: Spontaneously hypertensive rats
SHRSP: Spontaneously hypertensive stroke-prone rats
SPG: Sphenopalatine ganglion
STAIR: Stroke Therapy Academic Industry Roundtable
TIA: Transient ischemic attack
tMCAO: Transient middle cerebral artery occlusion
tPA: Tissue plasminogen activator
TPLSM: Two-photon laser scanning microscopy
VEGF-A/VEGFR2: Vascular endothelial growth factor A/B
VIP: Vasoactive intestinal polypeptide
WT: Wild-type
