# Correction to: Promising Cerebral Blood Flow Enhancers in Acute Ischemic Stroke

**DOI:** 10.1007/s12975-022-01110-8

**Published:** 2022-12-16

**Authors:** Ifechukwude Joachim Biose, Jadesola Oremosu, Somya Bhatnagar, Gregory Jaye Bix

**Affiliations:** 1 Department of Neurosurgery, Clinical Neuroscience Research Center, Tulane University School of Medicine, 131 S. Robertson, Ste 1300, Room 1349, New Orleans, LA 70112 USA; 2 School of Medicine, Tulane University, New Orleans, LA 70112 USA; 3 Tulane Brain Institute, Tulane University, New Orleans, LA 70112 USA; 4 Department of Neurology, Tulane University School of Medicine, New Orleans, LA 70112 USA; 5 Department of Microbiology and Immunology, Tulane University School of Medicine, New Orleans, LA 70112 USA; 6 School of Public Health and Tropical Medicine, Tulane University, New Orleans, LA 70122 USA

**Correction to: Translational Stroke Research**

**https://doi.org/10.1007/s12975-022-01100-w**

All authors' name read as last name, first name and middle name. For searchability and citation purposes, we would like the authors' name to read as first name, middle name and last name as follows:

Ifechukwude Joachim **Biose**, Jadesola **Oremosu**, Somya **Bhatnagar**, Gregory Jaye **Bix.**

We apologize for this error.

The original article has been corrected.

